# Receptor Contribution Reallocation After Removal of Literature-Derived Weights: A Secondary Sensitivity Analysis of the Integrated Ki-DDD-TWAS Pipeline for Antipsychotic Lipid-Trait Prioritization

**DOI:** 10.7759/cureus.113701

**Published:** 2026-07-30

**Authors:** Ngo Cheung, Hoi-Ki Cheung, Yee-Wah Yu, Yolanda Yuen-Ching Tsang

**Affiliations:** 1 Psychiatry, Cheung Ngo Medical Limited, Hong Kong, HKG; 2 Psychiatry, The Chinese University of Hong Kong, Hong Kong, HKG; 3 Psychiatry, The University of Hong Kong, Hong Kong, HKG

**Keywords:** adrb1, antipsychotics, defined daily dose, drug prioritization, hrh1, ki affinity, lipid traits, metabolic risk, receptor pharmacology, transcriptome-wide association study

## Abstract

Background and objective

Antipsychotic-associated metabolic abnormalities, including weight gain, dyslipidemia, hyperglycemia, obesity, and metabolic syndrome, vary among drugs. Receptor pharmacology offers mechanistic clues, while transcriptome-wide association studies identify genetically regulated expression signals linked to lipid traits. These evidence streams are usually evaluated separately. This secondary computational sensitivity analysis used previously generated Ki-DDD-TWAS outputs and was not a primary discovery or clinical validation study. The primary hypothesis was that removing literature-derived metabolic receptor weights would preserve overall drug ranking, with Spearman ρ approximately 0.9, while shifting receptor-level contributions from histaminergic and serotonergic systems toward dopaminergic and low-prior TWAS signals. Secondary objectives were to identify low-weight genes with strong TWAS signals, assess discordance with an author-defined clinical-liability comparator, and test sensitivity to gene removal and TWAS scaling.

Methods

Previously generated outputs were analyzed. Ki, the inhibition constant, and DDD, the defined daily dose, served as affinity and exposure-proxy inputs. Receptor-level and per-drug risk-score files were analyzed for 52 antipsychotics across LDL, HDL, log-transformed triglycerides, non-HDL, and total cholesterol. Analyses included reconstruction validation, pooled receptor contributions, TWAS signal versus literature weight, clinical-liability discordance, leave-one-gene-out and TWAS-scaling sensitivity, and receptor co-occurrence in high-risk drug sets. No patient-level data, independent TWAS resources, longitudinal outcomes, or outcome-based validation were used.

Results

Reconstruction reproduced saved risk ranks with Spearman correlation 1.000 for all 10 mode-trait combinations. V4 rankings remained concordant after removing literature weights, with trait-specific correlations of 0.900-0.923 and a mean of 0.913. The weighted model was dominated by HRH1, HTR2A, and HTR2C, contributing 25.7%, 21.6%, and 10.3% of pooled signal. Under uniform weighting, DRD2, DRD3, and HTR2A led, accounting for 22.1%, 12.9%, and 12.6%. ADRB1 was the strongest high-priority low-weight TWAS candidate, with an absolute HDL z-score of 13.53 and literature weight of 0.08. Other candidates included CHRM4, DRD2, DRD4, SLC6A4, and ADRB2. The model most strongly under-ranked paliperidone versus the supplied 12-drug comparator, while ziprasidone rose under uniform weighting. Removing HRH1 produced the largest rank shifts, particularly for levomepromazine, promazine, and acepromazine.

Conclusions

Removing literature-derived weights preserved overall drug ranking but substantially changed receptor-level score allocation. The weighted model emphasized histaminergic and serotonergic receptors prioritized in metabolic-liability literature, whereas the uniform model revealed broader dopaminergic and low-prior contributions. ADRB1 is a computational hypothesis-generating candidate, not a confirmed mechanism or established metabolic-risk contributor. HDL-association direction was not analyzed. The framework supports dual reporting of clinically anchored weighted and discovery-oriented uniform models as an internal sensitivity strategy only. It is not externally validated, does not predict patient-level outcomes, and should not be interpreted as estimating clinical metabolic risk or establishing receptor-level causality.

## Introduction

Clinical importance of antipsychotic metabolic heterogeneity

Antipsychotic medications are essential treatments for schizophrenia and other severe psychiatric disorders, but their use is associated with clinically important metabolic and cardiovascular complications. These complications include weight gain, abdominal adiposity, hyperglycemia, dyslipidemia, metabolic syndrome, and increased cardiovascular risk. The clinical consequences are particularly important because people with severe mental illness already experience substantial disparities in preventive care and cardiovascular health. Antipsychotic treatment therefore occurs within a population that may already have elevated baseline vulnerability to metabolic disease [1,2]. The present work addresses this clinical problem indirectly by evaluating whether a transparent computational prioritization framework can distinguish stable drug-level patterns from receptor-level assumptions.

The magnitude of metabolic liability is not uniform across drugs. Comparative clinical studies and network meta-analyses have demonstrated meaningful differences among individual antipsychotics, although the magnitude of those differences depends on the outcome assessed, treatment duration, study population, and comparator. In general, clozapine and olanzapine have been consistently associated with less favorable metabolic profiles, whereas several newer agents have shown lower average effects on weight or metabolic parameters in specific clinical settings [3,4]. These findings support the need for drug-level modeling rather than treating antipsychotic exposure as a homogeneous class effect.

Clinical heterogeneity is also evident across metabolic domains. A drug may show a strong association with weight gain but a less pronounced effect on a particular lipid trait, or may produce clinically relevant metabolic changes through mechanisms not captured by a single composite outcome. For this reason, the present study considered five related but nonidentical lipid traits: low-density lipoprotein cholesterol (LDL), high-density lipoprotein cholesterol (HDL), log-transformed triglycerides (log-TG), non-HDL, and total cholesterol (TC). These traits capture partially overlapping aspects of lipid biology and provide a broader basis for comparing drug-level patterns. Recent pharmacogenetic studies have also examined psychotropic-associated lipid changes using longitudinal clinical samples and genetic data, reinforcing the importance of separating lipid-specific hypotheses from broader metabolic outcomes [5]. 

Receptor pharmacology and metabolic liability

Receptor-binding pharmacology has long been used to explain differences in antipsychotic adverse effects. Histamine H1, serotonin 5-HT2A and 5-HT2C, alpha-adrenergic, muscarinic, and dopamine receptors have all been implicated in weight regulation, appetite, glucose metabolism, lipid metabolism, and antipsychotic-associated metabolic dysfunction [6-8]. A receptor-binding study reported that H1-histamine receptor affinity was correlated with short-term weight gain across typical and atypical antipsychotics, while alpha1A-adrenergic, 5-HT2C, and 5-HT6 receptor affinities also showed significant associations in its receptor panel [6].

These observations provide a biological rationale for assigning larger prior weights to receptors such as HRH1 and HTR2C. They do not, however, establish the exact numerical values used in the present weighting dictionary. Those values were investigator-defined computational priors based on an expert synthesis of receptor-binding and metabolic-liability literature; no formal consensus procedure or inter-rater reliability assessment was performed. Antipsychotics often interact with several receptor systems simultaneously, and the same drug can produce different downstream consequences depending on receptor occupancy, tissue distribution, intrinsic activity, dose, treatment duration, and host susceptibility. The “multisite” pharmacology of antipsychotics is therefore central to both their therapeutic effects and their adverse-effect profiles [9].

Receptor occupancy-based analyses have also shown that the apparent importance of a receptor depends not only on affinity but on exposure and the relationship between receptor occupancy and the clinical outcome being modeled [10]. Accordingly, an affinity-based score can be useful for comparative prioritization without being equivalent to a clinical risk estimate. The distinction is important for interpreting the results of the present analysis: a high computational contribution indicates that a receptor-gene feature contributes strongly to the model under the specified assumptions, not that the receptor independently causes metabolic disease.

In practical terms, a drug developer could use the two model modes to identify compounds whose computational score is driven mainly by receptors already associated with metabolic liability, such as H1 or 5-HT2C, versus compounds whose score is more dependent on lower-prior dopaminergic or adrenergic signals. Clinicians could use such information, if independently validated, to formulate pharmacological questions for monitoring or follow-up; the present scores are not intended to guide prescribing or treatment decisions. Recent precision-psychiatry literature emphasizes the need for transparent, biologically interpretable prioritization followed by independent validation rather than direct clinical implementation of unvalidated computational scores [11,12]. 

Transcriptome-wide association evidence

Transcriptome-wide association studies provide a complementary type of information. PrediXcan and related approaches use genetic variants to estimate the genetically regulated component of gene expression and then test its association with a phenotype [13]. Summary-statistic extensions allow transcriptome-wide associations to be calculated using genome-wide association study (GWAS) summary data, prediction weights, and linkage-disequilibrium information rather than individual-level genotype and phenotype data [14-17]. These approaches have been used to identify genes and tissues potentially involved in lipid traits, metabolic phenotypes, psychiatric disorders, and other complex traits.

The use of transcriptome-wide association study (TWAS) evidence introduces additional interpretive considerations. A TWAS association can arise when the predicted expression of a gene is correlated with a causal variant affecting the phenotype through another mechanism. Linkage disequilibrium, correlated expression-prediction models, tissue mismatch, and pleiotropy can therefore generate associations at genes that are not themselves causal. Fine-mapping and colocalization methods have been developed to address some of these issues, but a high TWAS statistic alone should not be treated as proof of causality [18,19].

Tissue context is another important consideration. Summary-based transcriptome analyses have shown that many associations are tissue-specific, while others appear in several tissues because of shared regulatory architecture or correlated prediction models [15]. These observations support broad, agnostic screening across available tissues, but they also make it difficult to infer the precise biological context of an association from a single summary statistic.

A further limitation is that the present model uses absolute z-scores and p-value-based scaling. Positive and negative gene-trait associations therefore contribute similarly to the computational score. In particular, a strong HDL association cannot be interpreted as harmful or protective without knowing the direction of the underlying association. A direction-aware sensitivity analysis was not performed and is a priority for future work.

Rationale for the present downstream analysis

The integrated Ki-DDD-TWAS (inhibition constant-defined daily dose-transcriptome-wide association study) pipeline was designed to combine pharmacological affinity, standardized dose information, and genetically informed lipid-trait associations. The upstream pipeline and its comparison of TWAS scaling methods have been described separately [20]. The current article is a secondary computational sensitivity analysis of those previously generated outputs. It does not introduce a new GWAS, retrain TWAS prediction models, or validate a clinical outcome model.

This question is distinct from asking whether the numerical scores increase or decrease. A weighting scheme can preserve the broad ranking while substantially changing which receptors account for the score. If literature weights emphasize known metabolic pathways, a weighted model may be clinically interpretable but partially circular. A uniform model removes that differential prior and allows receptor contribution to be determined by the underlying affinity, dose proxy, and TWAS information. Comparing the two modes therefore provides a sensitivity analysis for both ranking stability and model-attributed mechanistic attribution.

The analysis also addresses a practical problem in computational pharmacology. A drug can move in rank because its absolute score changes, because other drugs change more, or because a receptor-level component has been selectively amplified or suppressed. Receptor contribution analysis, candidate mapping, clinical discordance, and leave-one-gene-out testing help distinguish these possibilities.

Study objectives

The primary objective was to determine whether removal of literature-derived metabolic receptor weights alters drug ranking and receptor-level attribution in the V4 Ki-DDD-TWAS model. The primary hypothesis was that ranking would remain broadly stable, with Spearman ρ near 0.9, while receptor-level contributions would be reallocated from highly weighted histaminergic and serotonergic systems toward dopaminergic and other low-prior TWAS signals.

Secondary exploratory objectives were to (i) quantify changes in pooled receptor-gene contribution between the literature-weighted and uniform models; (ii) identify genes with strong lipid-related TWAS signals but low predefined literature weights; (iii) compare computational rankings with an author-defined 12-drug clinical-liability ordering, while recognizing that the two rankings contain different numbers of drugs and are not directly commensurate; (iv) assess sensitivity to removal of selected genes and to the transition from the V1 no-per-receptor-scaling implementation to V4 negative-log10-p-value scaling; and (v) define the appropriate hypothesis-generating and nonclinical interpretation of the weighted and uniform analyses.

## Materials and methods

Data sources and output files

This study used only previously generated pipeline outputs. The downstream analysis was generated on July 15, 2026, and loaded 30 of 30 expected input files. The primary V4 comparison used the identifiable long-format receptor-level file named n05a_ki_twas_full_results.csv and per-drug risk-score files generated under the V4 literature-weighted and V4 uniform roots. The supplied downstream record does not enumerate the complete filenames for all 30 inputs; this limits exact file-level reproducibility from the manuscript alone. The five traits were LDL, HDL, log-TG, non-HDL, and TC. The final analysis contained 52 drugs for each trait and each weighting mode.

Long-format files contained drug, receptor, mapped gene, Ki value, defined daily dose, affinity-dose score, TWAS z-score, TWAS p-value, strong-binder status, and TWAS-significance status. Per-drug files contained the final normalized model score, base score, maximum absolute TWAS z-score, number of nominally significant metabolic receptor rows, and risk rank. V1 risk-score files were additionally loaded for the comparison of no-scaling and V4 per-receptor scaling.

The pipeline’s broader upstream inputs included an N05A antipsychotic drug list, a Ki database, defined daily dose information [21], and precomputed lipid-related TWAS files. The exact source of the lipid GWAS/TWAS summary statistics, including the TWAS platform, tissue models, prediction-model reference panels, sample sizes, ancestry composition, and upstream quality-control procedures, was not preserved in the downstream file description supplied for this analysis. These details cannot be reconstructed without the upstream data and code archive and are therefore treated as a limitation rather than being inferred. The present downstream study did not retrain TWAS prediction models or reanalyze GWAS data. This distinction is important: the work evaluates a drug-level integration framework using existing TWAS outputs rather than introducing a new TWAS association method.

A reproducible release should include a versioned repository or supplementary archive containing the exact code, dependency versions for Python, pandas, NumPy, and SciPy, configuration files, input checksums, the complete file manifest, and a one-command reproduction script. The materials supplied for this manuscript do not establish that such an archive has already been deposited.

Reconstruction of V4 per-receptor contributions

The V4 implementation used the following reconstruction sequence. First, the affinity component was defined as the natural logarithm of one plus the nonnegative affinity-dose score. The affinity-dose score itself had been calculated upstream from inverse Ki multiplied by the logarithm of one plus the defined daily dose in milligrams.

For each receptor-gene row, the V4 TWAS scaling factor was calculated as the exponential of 0.045 multiplied by negative log10 of the TWAS p-value. Negative log10 p-values were capped at 25. When a p-value was missing, the scaling factor defaulted to 1 in the reconstruction. The receptor weight was the literature-derived metabolic weight in the weighted mode and 1.0 in the uniform mode.

The per-row contribution was therefore calculated as the transformed affinity multiplied by the receptor weight and the V4 TWAS scaling factor. Contributions were then summed within each drug to produce a reconstructed base score. The final drug score was reconstructed by multiplying the base score by one plus 0.02 multiplied by the drug’s maximum absolute TWAS z-score and by one plus 0.10 multiplied by the number of nominally significant metabolic receptor rows. Scores were normalized to chlorpromazine, which was set to 100.

The coefficients 0.045, 0.02, and 0.10, together with the p-value cap of 25, were heuristic choices intended to provide numerical stability and graded rather than binary boosts. They were not optimized against clinical outcomes, calibrated to observed lipid changes, or selected using an independent validation set. A formal coefficient-range sensitivity analysis was not available in the supplied downstream outputs; no claim of coefficient robustness is therefore made.

The reconstruction was intentionally limited to the formulas used in the V4 scoring implementation. It was not intended to establish that the formula is biologically correct. Rather, it tested whether the downstream contribution calculations reproduced the saved output ranks. A reconstruction correlation of 1.000 confirms internal implementation consistency only; it does not establish biological validity, clinical calibration, or predictive validity.

Literature-weighted and uniform models

The literature-weight dictionary assigned the greatest prior weights to HRH1, HTR2C, and CHRM3, with values of 1.00, 0.92, and 0.85, respectively. HTR2A received a weight of 0.55; ADRA1A and ADRA1B received 0.48; HTR6 received 0.42; ADRA2A, ADRA2B, and ADRA2C received 0.32, 0.30, and 0.30; CHRM1 received 0.28; HTR7 received 0.25; CHRM4 received 0.22; CHRM5 received 0.20; and DRD3 received 0.18. Lower weights were assigned to DRD2, DRD4, HTR1A, ADRB1, ADRB2, SIGMAR1, SLC6A4, SLC6A2, and SLC6A3.

The weights were assigned by the investigator's synthesis of receptor-binding and metabolic-liability literature (Appendix 1). No formal Delphi process, independent replication of the dictionary, or inter-rater agreement assessment was performed. The complete weight dictionary and the supporting citation for every non-zero weight should accompany the code and supplementary data. The numerical weights should therefore be interpreted as transparent but subjective priors.

In the uniform model, all recognized receptor genes contributed with a weight of 1.0. The uniform mode was not treated as an absence of pharmacology or TWAS information. It retained the same Ki, dose, receptor mapping, TWAS scaling, secondary drug-level multiplier, and normalization procedure. Only the differential receptor weights were removed.

Missingness and imputation

The supplied downstream summary tables do not contain row-level flags identifying observed Ki values, imputed Ki values, observed TWAS values, imputed or default TWAS values, or the number of rows using the default scaling factor of 1. Aggregate contribution and rank tables cannot be used to reconstruct those flags without the underlying long-format files and imputation logs. Accordingly, exact counts by drug, receptor, and trait are not reported here.

The pipeline description refers to mean Ki imputation, TWAS imputation, and default handling of missing p-values, but the supplied outputs do not permit independent quantification of their frequency. This is a reproducibility limitation because a drug’s position may be affected by imputed rather than directly observed values. A complete row-level missingness table should be generated from the underlying files before the framework is considered for external validation.

Global receptor contribution analysis

For each trait and weighting mode, reconstructed per-row contributions were summed by receptor and gene. Because several receptor labels could map to the same official gene symbol, pooled contributions across traits were aggregated at the gene level. The contribution of each gene was expressed both as an absolute pooled contribution and as a percentage of the total pooled signal within the relevant weighting mode.

This analysis was intended to identify whether the two models emphasized the same biological systems. It was not interpreted as an estimate of the fraction of clinical metabolic risk mediated by a particular receptor.

TWAS signal versus literature weight

For each gene, the largest observed absolute TWAS z-score across the five traits was identified. The trait in which the maximum occurred was retained as the peak trait. Each gene was then assigned its literature weight from the predefined dictionary; genes absent from the dictionary received a weight of zero.

Genes were classified as novel-mechanism candidates when their maximum absolute TWAS z-score was at least 4.0 and their literature weight was no greater than 0.30. This was an exploratory post hoc prioritization rule, not a prespecified confirmatory threshold. It did not represent a genome-wide significance threshold, false-discovery-rate control, Bonferroni correction, colocalization evidence, or a causal criterion.

The candidate analysis was based on the maximum absolute z-score across traits. It therefore prioritized strong gene-trait signals but did not account for cross-trait dependence, direction consistency, tissue specificity, colocalization, or the number of independent variants contributing to the association. Because the model uses absolute z-scores and p-values, the candidate rule cannot determine whether a signal is associated with higher or lower HDL, LDL, triglycerides, or other lipid traits.

Clinical discordance analysis

The analysis compared the mean model rank across the five traits with a clinical-liability ordering encoded in the supplied Stage 2 code. The comparator assigned ranks from 1 to 12 to clozapine, olanzapine, quetiapine, paliperidone, risperidone, asenapine, aripiprazole, amisulpride, ziprasidone, lurasidone, haloperidol, and cariprazine.

The model rank was obtained by averaging risk ranks across the five lipid traits and then re-ranking the drugs. Discordance was calculated as model rank minus clinical rank. A positive value indicated that the model assigned a numerically lower-risk position than the comparator, whereas a negative value indicated that the model assigned a higher-risk position.

The comparator was an author-defined reference ordering encoded in the analysis and was not derived from a single cited network meta-analysis or a single outcome-specific clinical dataset. The exact source papers used to construct the 12-drug order were not retained in the supplied downstream archive. The comparator should therefore be regarded as a descriptive reference ordering, not a gold-standard clinical outcome ranking.

This analysis was not a clinical validation study. The comparator was treated as an external reference ordering specified in the analysis code, not as an infallible gold standard. No patient-level outcomes, event rates, or prospective clinical predictions were evaluated. Because the comparator contains 12 drugs and the computational model ranks 52 drugs, absolute rank differences are not on the same scale. The discordance analysis is therefore hypothesis-triage only and should not be interpreted as a direct test of the model’s clinical accuracy.

Leave-one-gene-out sensitivity analysis

The genes HRH1, HTR2C, CHRM3, and ADRB1 were removed individually from the reconstructed V4 contribution tables. Drug scores and ranks were recalculated for each of the five traits and compared with the corresponding full model.

The rank shift was defined as the rank after gene removal minus the baseline rank. A positive shift indicated that the drug moved toward a numerically lower-risk position after removal. Mean shifts were then calculated across traits. The analysis measured model dependence on each gene; it did not establish that a gene was biologically causal.

TWAS-scaling sensitivity

The V1 implementation used no per-receptor TWAS scaling but retained its drug-level maximum-absolute-z multiplier. V4 applied exponential scaling based on the negative log10 of the TWAS p-value. The difference between the V1 rank and V4 rank was calculated for each drug and trait. Positive values indicated that V4 scaling moved a drug toward a higher-risk position relative to V1.

This comparison was interpreted as a sensitivity analysis of the TWAS transformation, separate from the comparison of literature-weighted and uniform modes.

Statistical analysis and reproducibility

Rank agreement was evaluated using Spearman and Kendall correlations. The primary interpretation focused on Spearman correlation because the central question was whether drug ordering was preserved. Pearson correlation between the base score and final score was used to estimate how strongly the final risk index remained aligned with the pre-TWAS score component.

Confidence intervals for the rank correlations and formal statistical comparisons between the V1 and V4 correlation means were not available in the supplied summary output. The difference between the V1 and V4 means is therefore treated as descriptive rather than statistically tested. Similarly, score-level reconstruction statistics, including mean absolute error and root mean square error, were not calculated from the supplied tables.

All calculations were implemented in Python using pandas and NumPy, with SciPy used for correlation statistics. The Stage 2 code also used Matplotlib for visualization. The analysis was run from saved output files, and reconstruction validation was based on direct comparison between reconstructed and saved risk ranks.

This study is not a clinical prediction-model development or validation study. Full TRIPOD+AI reporting is therefore not directly applicable, although relevant transparency items concerning model specification, intended use, predictors, missing data, performance, and limitations are addressed [22,23]. Reporting of the genetic-association component was considered in light of STREGA principles [22].

Code availability

The analysis code is publicly available in the GitHub repository 080JvE-Receptor-Contribution-Reallocation-After-Removal-of-Literature-Derived-Weights. The repository contains the notebook 080JvE(compare_without_weights)_TWAS_Lipids_Risk_x_Antipsychotics_Ki.ipynb and a README describing the project.

The code is notebook-based and includes the analyses comparing receptor contribution patterns after removal of literature-derived weights. Complete reproduction additionally requires the relevant input files, configuration information, dependency versions, receptor-weight dictionary, imputation records, and any upstream TWAS files used to generate the analyzed outputs. Accordingly, the repository provides the principal analysis code but should not be interpreted as a complete self-contained reproduction archive unless these additional materials are deposited.

**Figure 1:**
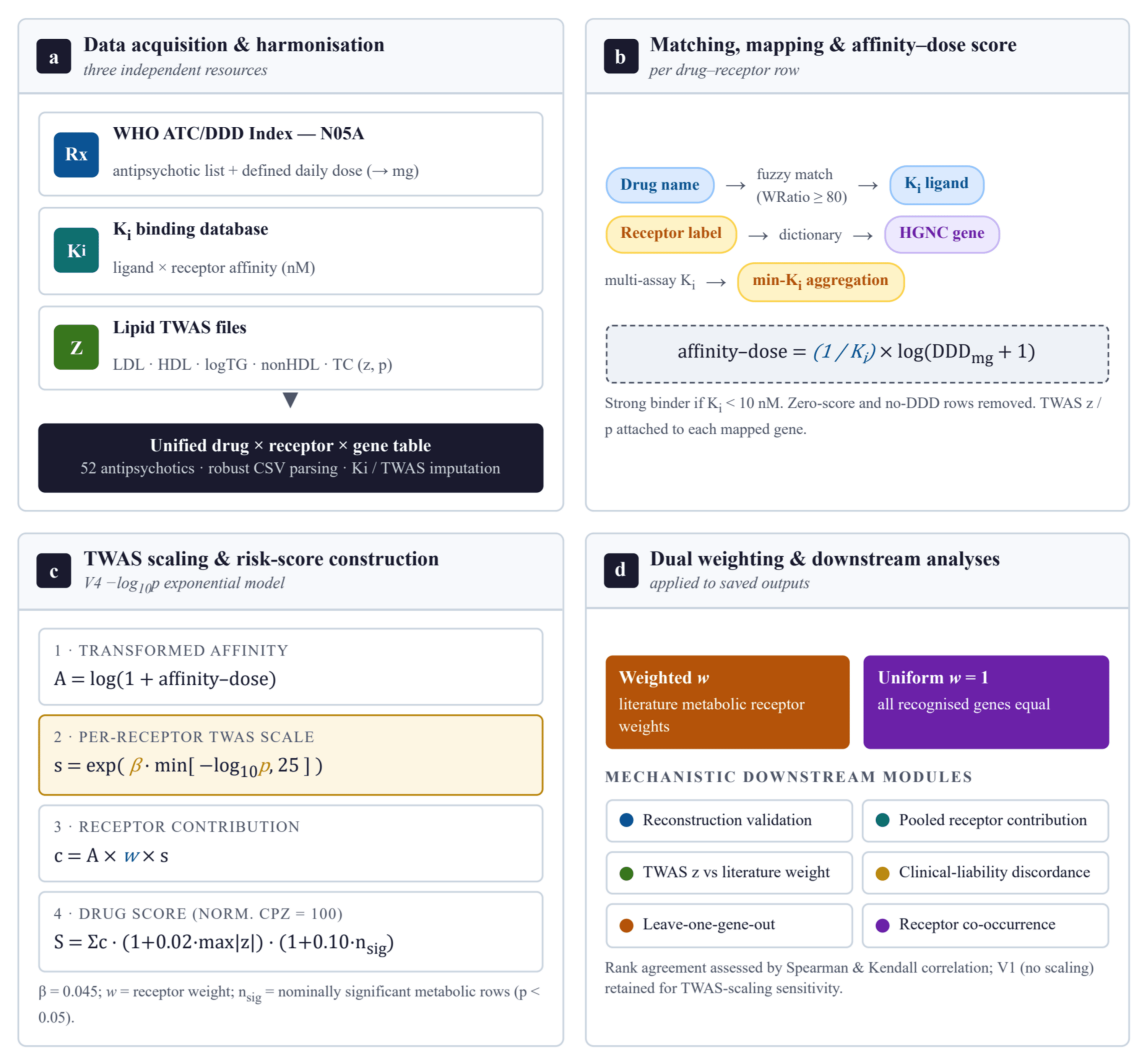
Integrated Ki-DDD-TWAS pipeline for antipsychotic metabolic-risk modeling Schematic of the four-stage analytical workflow. (a) Harmonization of three independent data sources. (b) Fuzzy drug-ligand matching, receptor-to-gene mapping, and affinity-dose scoring. (c) Per-receptor transcriptome-wide scaling and drug-level score construction. (d) Dual literature-weighted versus uniform-weight scoring and the downstream sensitivity analyses applied to the saved outputs. The workflow is a secondary computational prioritization framework; no patient-level outcome prediction is performed. The pipeline consumes pre-computed TWAS summary statistics; no GWAS reanalysis or model retraining is performed. Credits: Ngo Cheung ATC, anatomical therapeutic chemical; DDD, defined daily dose; Ki, inhibition constant; TWAS, transcriptome-wide association study; GWAS, genome-wide association study; CPZ, chlorpromazine; LDL/HDL, low-/high-density lipoprotein cholesterol; logTG, log-transformed triglycerides; TC, total cholesterol

Figure 1 visualizes the entire Ki-DDD-TWAS pipeline for antipsychotic metabolic-risk modeling.

## Results

Input coverage and reconstruction fidelity

All 30 expected Stage 2 input files were located and readable (Table 1). The V4 analysis covered 52 drugs across five lipid traits in both the literature-weighted and uniform modes. The reconstructed risk ranks matched the saved ranks with a Spearman correlation of 1.000 for LDL, HDL, log-TG, non-HDL, and TC in both modes. The mean Spearman correlation across all 10 comparisons was also 1.000.

**Table 1:** Reconstruction validation Note: This table evaluates internal rank-reconstruction fidelity. Absolute-score agreement metrics, including Pearson correlation, mean absolute error, and root mean square error, were not available from the supplied downstream output and are not inferred from Spearman correlation. A perfect rank correlation does not establish biological or clinical validity. LDL/HDL, low-/high-density lipoprotein cholesterol; logTG, log-transformed triglycerides; TC, total cholesterol

Mode	Trait	n	Spearman ρ with saved risk rank
V4 with-weights	LDL	52	1.000
V4 with-weights	HDL	52	1.000
V4 with-weights	log-TG	52	1.000
V4 with-weights	non-HDL	52	1.000
V4 with-weights	TC	52	1.000
V4 uniform	LDL	52	1.000
V4 uniform	HDL	52	1.000
V4 uniform	log-TG	52	1.000
V4 uniform	non-HDL	52	1.000
V4 uniform	TC	52	1.000

This result confirms that the downstream contribution reconstruction reproduced the ranking behavior of the saved V4 outputs under the supplied formulas. It does not demonstrate external validity, clinical calibration, causal validity, or patient-level predictive performance. It also does not prove that the reconstructed absolute scores were identical in every numerical detail; the reported validation statistic concerned rank agreement only.

Overall rank concordance after removal of literature weights

The V4 ranking remained broadly stable after the literature weights were removed. Trait-specific Spearman correlations between the weighted and uniform models were 0.902 for LDL, 0.923 for HDL, 0.917 for log-TG, 0.900 for non-HDL, and 0.920 for TC. The mean Spearman correlation was 0.913. Corresponding Kendall correlations ranged from 0.768 to 0.801, with a mean of 0.786.

The V1 comparison was slightly more concordant, with a mean Spearman correlation of 0.927 and a mean Kendall correlation of 0.802. The difference between the V1 and V4 means was small and was not formally tested; it is therefore interpreted descriptively rather than as evidence that V4 is statistically more sensitive to weight removal. The V4 ranking nevertheless remained highly concordant after removal of literature weights (Table 2).

**Table 2:** Weighted-versus-uniform rank concordance Note: Risk rank 1 denotes the highest model-prioritization position. The positive rank-change convention is stated explicitly in the leave-one-gene-out and discordance analyses. Confidence intervals and formal comparisons of V1 and V4 correlation estimates were not available in the supplied summary output. LDL/HDL, low-/high-density lipoprotein cholesterol; logTG, log-transformed triglycerides; TC, total cholesterol

Version	Trait	n	Spearman ρ	Kendall τ
V1_original	LDL	52	0.919	0.784
V1_original	HDL	52	0.949	0.831
V1_original	Log-TG	52	0.926	0.802
V1_original	Non-HDL	52	0.919	0.792
V1_original	TC	52	0.924	0.802
V1_original	Mean	-	0.927	0.802
V4_logp_expo	LDL	52	0.902	0.771
V4_logp_expo	HDL	52	0.923	0.801
V4_logp_expo	Log-TG	52	0.917	0.793
V4_logp_expo	Non-HDL	52	0.900	0.768
V4_logp_expo	TC	52	0.920	0.799
V4_logp_expo	Mean	-	0.913	0.786

The stability was strongest at the top of the ranking. Clozapine remained ranked first across all traits in both modes. Chlorpromazine remained the normalization reference and was generally ranked second, although ziprasidone ranked second and chlorpromazine third in the uniform HDL analysis. Asenapine, zotepine, risperidone, ziprasidone, olanzapine, and thioridazine occupied the remaining high-ranking positions with moderate reshuffling.

Clozapine therefore remained the most stable high-priority drug in this computational framework, whereas ziprasidone showed the largest upward movement among the leading drugs under uniform weighting. Removal of differential weights also reduced the relative positions of several drugs with strong H1-related contributions, particularly phenothiazine-related agents. These are observations about model behavior and should not be interpreted as patient-level clinical predictions.

The cross-trait V4 consensus ranking placed clozapine first with a mean rank of 1.00; chlorpromazine second with a mean rank of 2.00; asenapine third with a mean rank of 3.00; zotepine fourth with a mean rank of 4.40; risperidone fifth with a mean rank of 4.80; ziprasidone sixth with a mean rank of 5.80; olanzapine seventh with a mean rank of 7.00; and thioridazine eighth with a mean rank of 8.00 in the weighted model. Under uniform weighting, clozapine remained first, but ziprasidone moved to a mean rank of 2.80, followed by chlorpromazine at 2.20, asenapine at 4.20, risperidone at 4.80, zotepine at 6.00, thioridazine at 7.00, and haloperidol at 8.60.

Mean rank was used because it provides a simple cross-trait summary on the same ordinal scale. Median-rank and Borda-count alternatives were not evaluated in the supplied outputs, so similarity of those alternatives is not claimed. The resulting table is a lipid-trait-based composite ranking, not a comprehensive metabolic-risk ranking incorporating weight, glucose, diabetes, or metabolic-syndrome outcomes (Table 3).

**Table 3:** Cross-trait lipid-trait-based composite ranking Note: Mean scores are normalized to chlorpromazine = 100. The table summarizes five lipid traits only and should not be interpreted as a validated clinical metabolic-risk ranking.

Version	Weight mode	Rank	Drug	Mean rank	Mean score	Rank range
V4_logp_expo	With-weights	1	Clozapine	1.00	121.4	1-1
V4_logp_expo	With-weights	2	Chlorpromazine	2.00	100.0	2-2
V4_logp_expo	With-weights	3	Asenapine	3.00	85.1	3-3
V4_logp_expo	With-weights	4	Zotepine	4.40	76.2	4-6
V4_logp_expo	With-weights	5	Risperidone	4.80	73.9	4-5
V4_logp_expo	With-weights	6	Ziprasidone	5.80	72.7	5-6
V4_logp_expo	With-weights	7	Olanzapine	7.00	67.3	7-7
V4_logp_expo	With-weights	8	Thioridazine	8.00	62.7	8-8
V4_logp_expo	With-weights	9	Chlorprothixene	9.20	54.9	9-10
V4_logp_expo	With-weights	10	Sertindole	9.80	48.5	9-10
V4_logp_expo	Uniform	1	Clozapine	1.00	114.7	1-1
V4_logp_expo	Uniform	2	Chlorpromazine	2.20	100.0	2-3
V4_logp_expo	Uniform	3	Ziprasidone	2.80	97.1	2-3
V4_logp_expo	Uniform	4	Asenapine	4.20	91.0	4-5
V4_logp_expo	Uniform	5	Risperidone	4.80	89.2	4-5
V4_logp_expo	Uniform	6	Zotepine	6.00	77.5	6-6
V4_logp_expo	Uniform	7	Thioridazine	7.00	74.8	7-7
V4_logp_expo	Uniform	8	Haloperidol	8.60	59.4	8-9
V4_logp_expo	Uniform	9	Chlorprothixene	9.80	56.0	8-13
V4_logp_expo	Uniform	10	Iloperidone	10.40	55.7	10-11

These results indicate that removal of literature weights did not eliminate the broad model-prioritization signal. Instead, it changed the relative position of several drugs, especially those in the middle and upper-middle portions of the ranking.

Reallocation of global receptor contributions

The clearest model-attribution difference between the two modes was observed in the pooled receptor contributions. In the literature-weighted model, HRH1 was the largest contributor, accounting for 25.7% of the total pooled signal. HTR2A contributed 21.6%, and HTR2C contributed 10.3%. Together, these three genes accounted for 57.6% of the pooled contribution. DRD2 contributed 8.3%, DRD3 contributed 7.3%, and ADRA1A contributed 6.3%.

The remaining leading weighted contributors were HTR7 at 3.8%, ADRA1B at 3.7%, HTR6 at 3.5%, and DRD4 at 2.7%. CHRM3 contributed 1.2% of the pooled weighted signal despite receiving a high prior weight of 0.85. This distinction is important: a high predefined weight does not guarantee a large empirical contribution after the affinity, dose, and TWAS components are applied.

The uniform model produced a different contribution structure. DRD2 became the largest contributor at 22.1%, followed by DRD3 at 12.9% and HTR2A at 12.6%. DRD4 contributed 8.8%, and HRH1 accounted for 8.2% of the total. HTR7 contributed 4.9%, ADRA1A 4.2%, HTR2B 3.7%, HTR2C 3.6%, and HTR6 2.7%.

The fall in HRH1’s percentage contribution did not reflect a fall in its absolute contribution. HRH1 contributed 153.4 pooled units in both modes; its percentage decreased because the uniform model expanded the denominator by increasing the contributions of lower-prior receptors (Table 4). This distinction is central to interpreting the reallocation.

**Table 4:** Global per-receptor contribution pooled across traits Note: The table shows the top 15 genes separately for each weighting mode. The complete receptor list and full per-trait contribution matrices should be supplied as supplementary data. Absolute contributions and percentages should be considered model quantities, not estimates of clinical mediation.

With-weights gene	Contribution	% total	Uniform gene	Contribution	% total
HRH1	153.4	25.7	DRD2	412.1	22.1
HTR2A	129.2	21.6	DRD3	241.0	12.9
HTR2C	61.34	10.3	HTR2A	234.8	12.6
DRD2	49.45	8.3	DRD4	163.8	8.8
DRD3	43.37	7.3	HRH1	153.4	8.2
ADRA1A	37.68	6.3	HTR7	91.48	4.9
HTR7	22.87	3.8	ADRA1A	78.51	4.2
ADRA1B	22.29	3.7	HTR2B	68.73	3.7
HTR6	21.04	3.5	HTR2C	66.68	3.6
DRD4	16.38	2.7	HTR6	50.10	2.7
ADRA2C	7.876	1.3	ADRA1B	46.44	2.5
CHRM1	7.299	1.2	HTR1A	39.10	2.1
CHRM3	6.969	1.2	ADRA2C	26.25	1.4
ADRA2B	6.349	1.1	CHRM1	26.07	1.4
HTR1A	3.128	0.5	ADRA2B	21.16	1.1

The absolute pooled contribution of DRD2 increased from 49.45 under the weighted model to 412.1 under uniform weighting. DRD3 increased from 43.37 to 241.0, and DRD4 increased from 16.38 to 163.8. These changes reflect the removal of differential weighting rather than a change in the underlying Ki or TWAS data. Conversely, HRH1 retained a similar absolute contribution in both modes because its weight was already 1.0, but its percentage contribution decreased as other receptors gained relative importance.

The weighted model therefore produced a predominantly histaminergic and serotonergic model-attribution profile, with substantial contributions from dopamine and adrenergic receptors. The uniform model produced a broader pharmacological profile in which dopamine receptor engagement, particularly DRD2, DRD3, and DRD4, became more prominent. This was a reallocation of the model’s receptor-level explanation, not evidence that dopaminergic receptors account for a corresponding proportion of clinical lipid risk.

TWAS signal and low-weight candidate genes

The TWAS-versus-literature-weight analysis identified six genes that met the exploratory candidate criteria of an absolute z-score of at least 4.0 and a literature weight of no more than 0.30. These candidates are listed in Table 5.

**Table 5:** Exploratory low-weight genes with strong TWAS signals Note: Candidates were defined using an exploratory post hoc rule of a maximum absolute z-score of at least 4.0 and a literature weight no greater than 0.30. No FDR or Bonferroni correction, replication analysis, colocalization, or fine-mapping was performed. TWAS, transcriptome-wide association study; LDL/HDL, low-/high-density lipoprotein cholesterol; logTG, log-transformed triglycerides; TC, total cholesterol; FDR, false discovery rate

Gene	Maximum absolute TWAS z-score	Peak trait	Literature weight	Exploratory candidate
ADRB1	13.53	HDL	0.08	Yes
CHRM4	6.49	TC	0.22	Yes
DRD2	4.71	log-TG	0.12	Yes
DRD4	4.39	log-TG	0.10	Yes
SLC6A4	4.08	log-TG	0.06	Yes
ADRB2	4.07	log-TG	0.07	Yes

ADRB1 was the strongest computational candidate. Its maximum absolute TWAS z-score was 13.53 for HDL, while its literature weight was 0.08. In the significant-hit output, the corresponding p-value was approximately 1 × 10⁻⁴¹. ADRB1 therefore had an unusually strong genetic association signal relative to its low prior weight.

The HDL association cannot be interpreted as harmful or protective because the model retained only the absolute association strength and not the direction of effect. CHRM4 was the second-ranked candidate, with an absolute z-score of 6.49 for TC and a literature weight of 0.22. DRD2 had an absolute z-score of 4.71 for log-TG and a weight of 0.12. DRD4 had an absolute z-score of 4.39 for log-TG and a weight of 0.10. SLC6A4 had an absolute z-score of 4.08 for log-TG and a weight of 0.06, while ADRB2 had an absolute z-score of 4.07 for log-TG and a weight of 0.07.

These genes were identified as underweighted candidates, not as confirmed new mechanisms. The ADRB1 signal was particularly prominent because the same gene-level HDL association was paired with multiple drugs that had ADRB1 records. ADRB1 appeared in 20 drug-level significant-hit records, but these appearances should not be interpreted as 20 independent genetic replications. They represent multiple pharmacological exposures being evaluated against the same underlying gene-trait signal.

The supplied summary output does not identify whether every low z-value was observed, imputed, or assigned through a default procedure. In particular, the HTR2C value of approximately 0.42 should not be interpreted as evidence for or against a strong lipid TWAS association without row-level missingness and imputation flags.

Discordance with the clinical-liability comparator

The weighted V4 model showed the largest positive discordance for paliperidone. Its clinical comparator rank was 4, whereas its model rank was 35, producing a discordance of +31. Amisulpride had a clinical rank of 8 and a model rank of 28, giving a discordance of +20. Lurasidone and cariprazine each had discordances of +14, while quetiapine had a discordance of +11. Aripiprazole had a discordance of +12.

The weighted model assigned numerically more risk-oriented positions to several drugs than the comparator. Asenapine and ziprasidone were each assigned a discordance of -3, meaning that their model positions were numerically more risk-oriented than their comparator positions. Clozapine and risperidone had no discordance in the weighted comparison.

Uniform weighting increased some of the positive disagreements. Paliperidone moved to model rank 41 against comparator rank 4, producing a discordance of +37. Quetiapine moved to rank 23 against comparator rank 3, producing a discordance of +20. Lurasidone had a discordance of +16, and amisulpride had a discordance of +14. Aripiprazole had a discordance of +7, while cariprazine had a discordance of +5.

At the same time, uniform weighting increased the relative prominence of ziprasidone and haloperidol. Ziprasidone reached model rank 3 against comparator rank 9, a discordance of -6. Haloperidol reached rank 8 against comparator rank 11, a discordance of -3 (Table 6).

**Table 6:** Descriptive discordance between model rankings and the author-defined clinical-liability comparator Note: Discordance was calculated as model rank minus clinical rank. Positive values indicate that the model assigned a numerically lower-risk position than the comparator. The comparator contains 12 drugs, whereas the model ranks 52; the numerical differences are therefore not directly commensurate and should not be interpreted as validated clinical prediction errors.

Drug	Clinical rank	V4 weighted model rank	Weighted discordance	V4 uniform model rank	Uniform discordance
Clozapine	1	1.0	0	1.0	0
Olanzapine	2	7.0	+5	11.2	+10
Quetiapine	3	14.0	+11	22.6	+20
Paliperidone	4	35.0	+31	41.2	+37
Risperidone	5	4.8	0	4.8	0
Asenapine	6	3.0	-3	4.2	-2
Aripiprazole	7	19.2	+12	14.0	+7
Amisulpride	8	29.0	+20	21.4	+14
Ziprasidone	9	5.8	-3	2.8	-6
Lurasidone	10	24.6	+14	26.6	+16
Haloperidol	11	17.4	+6	8.6	-3
Cariprazine	12	25.6	+14	17.4	+5

These discrepancies should not be interpreted as evidence that the clinical comparator is wrong or that the computational model has discovered previously unrecognized clinical risk. They indicate that the computational score and the comparator are measuring different constructs. Potential explanations include incomplete receptor coverage, the use of defined daily dose as an exposure proxy, minimum-Ki aggregation, receptor subtype simplification, missing or imputed TWAS values, active metabolites, and the absence of clinical outcome calibration.

In particular, the paliperidone and ziprasidone comparisons should not be given biological meaning until the comparison is recalculated using identical drug sets and a cited, outcome-specific comparator. In the present analysis, these findings are hypothesis-triage signals only.

Leave-one-gene-out sensitivity

HRH1 was the dominant model driver in the weighted analysis. Removing HRH1 increased the mean rank of levomepromazine by 31.2 positions, promazine by 19.8 positions, and acepromazine by 19.2 positions. Tiotixene shifted by 8.6 positions, quetiapine by 8.2, and mesoridazine by 5.8. A positive shift indicates movement toward a numerically lower-risk position after HRH1 was removed.

The corresponding uniform analysis showed smaller but still detectable HRH1 dependence. Levomepromazine shifted by 12.6 positions, quetiapine by 7.4, acepromazine by 5.2, promazine by 4.4, loxapine by 3.2, and mesoridazine by 3.0. These results confirm that HRH1 was not simply a high-ranked receptor in the contribution table; it directly influenced the relative positions of drugs with strong H1-related contributions.

Removing HTR2C produced more moderate shifts. In the weighted model, asenapine shifted by 3.8 positions, ziprasidone by 2.6, and sertindole by 1.8. In the uniform model, loxapine and sertindole each shifted by 1.4 positions, while ziprasidone and asenapine shifted by 1.0 and 0.8 positions, respectively. These effects suggest that HTR2C contributed to the ranking but that its influence was distributed across several drugs.

CHRM3 removal had little overall effect. The largest weighted shift was observed for haloperidol, with a mean change of -0.4 positions, while loxapine and mesoridazine changed by approximately +0.2 positions. Under uniform weighting, the largest shifts were +0.6 for mesoridazine and +0.4 for loxapine. Thus, CHRM3 had a high predefined weight but relatively limited leverage in the observed drug-ranking structure.

ADRB1 removal generated smaller shifts than HRH1 removal but provided evidence that the strong ADRB1 TWAS signal entered the score in a measurable way. In the weighted model, amisulpride shifted by +2.8 positions and fluspirilene by +2.2. Quetiapine shifted by +0.8. In the uniform model, loxapine shifted by +2.0, quetiapine by +1.8, and amisulpride and fluspirilene by +1.0. These findings support ADRB1 as a computationally influential candidate, but they do not demonstrate biological causality.

Only mean shifts across five traits were available in the supplied summary (Table 7). Standard deviations, ranges, trait-specific shift distributions, and bootstrap uncertainty estimates were not available and are therefore not reported.

**Table 7:** Selected largest mean rank shifts after leave-one-gene-out analysis A positive shift indicates movement toward a numerically lower-risk position after removal. The table reports mean shifts only; no significance test or uncertainty interval was available.

Removed gene	Mode	Drug	Mean rank shift	n traits
HRH1	With-weights	Levomepromazine	+31.2	5
HRH1	With-weights	Promazine	+19.8	5
HRH1	With-weights	Acepromazine	+19.2	5
HRH1	Uniform	Levomepromazine	+12.6	5
HRH1	Uniform	Quetiapine	+7.4	5
HRH1	Uniform	Acepromazine	+5.2	5
HTR2C	With-weights	Asenapine	+3.8	5
HTR2C	With-weights	Ziprasidone	+2.6	5
HTR2C	With-weights	Sertindole	+1.8	5
HTR2C	Uniform	Loxapine	+1.4	5
HTR2C	Uniform	Sertindole	+1.4	5
HTR2C	Uniform	Ziprasidone	+1.0	5
CHRM3	With-weights	Haloperidol	-0.4	5
CHRM3	With-weights	Loxapine	+0.2	5
CHRM3	With-weights	Mesoridazine	+0.2	5
CHRM3	Uniform	Mesoridazine	+0.6	5
CHRM3	Uniform	Loxapine	+0.4	5
CHRM3	Uniform	Amisulpride	-0.2	5
ADRB1	With-weights	Amisulpride	+2.8	5
ADRB1	With-weights	Fluspirilene	+2.2	5
ADRB1	With-weights	Quetiapine	+0.8	5
ADRB1	Uniform	Loxapine	+2.0	5
ADRB1	Uniform	Quetiapine	+1.8	5
ADRB1	Uniform	Amisulpride	+1.0	5

TWAS-scaling sensitivity, TWAS influence, and receptor co-occurrence

The transition from V1 to V4 produced smaller changes than the removal of literature weights for most leading drugs. In the weighted mode, the largest mean effects were observed for fluspirilene, which shifted by -3.8 positions; cariprazine, -3.4; bromperidol, -2.8; perphenazine, +2.2; pipamperone, -2.0; melperone, -1.8; and amisulpride, -1.8. In the uniform mode, pipamperone shifted by -3.2, loxapine by -2.8, fluspirilene by -2.6, chlorprothixene by +2.6, amisulpride by -2.2, moperone by +2.0, and trifluoperazine by +2.0.

The high Pearson correlations between base score and final risk score also indicated that TWAS scaling acted primarily as a refinement. In V4, correlations ranged from 0.988 to 0.996 in the weighted mode and from 0.978 to 0.993 in the uniform mode. The lower correlations occurred for HDL, particularly under uniform weighting, but the overall relationship remained strong. The V1 correlations ranged from 0.962 to 0.992.

These correlations describe the relationship between two components of the computational score. They should not be interpreted as evidence that TWAS scaling improves clinical prediction.

Receptor co-occurrence in the top-ten high-risk drugs showed frequent combinations in both modes. In the weighted mode, several pairs reached the maximum pooled count of 50 drug-trait occurrences, including DAT-M3, 5-HT2A-D4, 5-HT2C-M1, 5-HT2C-M3, 5-HT2C-D1, 5-HT2C-D2, 5-HT2C-D3, 5-HT2C-D4, 5-HT1A-M1, 5-HT1A-M3, 5-HT2A-M1, and 5-HT2A-M3. In the uniform mode, frequently occurring pairs included 5-HT1A with 5-HT3, 5-HT6, 5-HT7, 5-HT2A, M1, 5-HT2C, D1, and H1, as well as Beta2-D1 and M1-M3.

Because the maximum count was 50, corresponding to ten top drugs across five traits, these co-occurrence values were partly determined by the stability of the top-10 drug sets. They should not be interpreted as independent interaction evidence or as statistical evidence of receptor synergy.

## Discussion

Principal mechanistic insight

The central finding of this secondary computational sensitivity analysis was that removal of literature-derived receptor weights preserved broad drug ranking while changing the model-attributed receptor composition. The V4 weighted model emphasized HRH1, HTR2A, and HTR2C, while the uniform model emphasized DRD2, DRD3, DRD4, and HTR2A. The resulting mean Spearman correlation of 0.913 indicates substantial preservation of rank order, but it does not imply mechanistic equivalence.

This distinction is important. A model may be robust at the level of drug prioritization while remaining sensitive at the level of biological explanation. Clozapine’s first-place position was stable, but the receptor pathways accounting for the score differed considerably. In the weighted model, the prior structure concentrated signal in receptor systems already associated with metabolic effects. In the uniform model, receptor contribution was determined more directly by the combination of affinity, dose proxy, and TWAS scaling.

**Figure 2:**
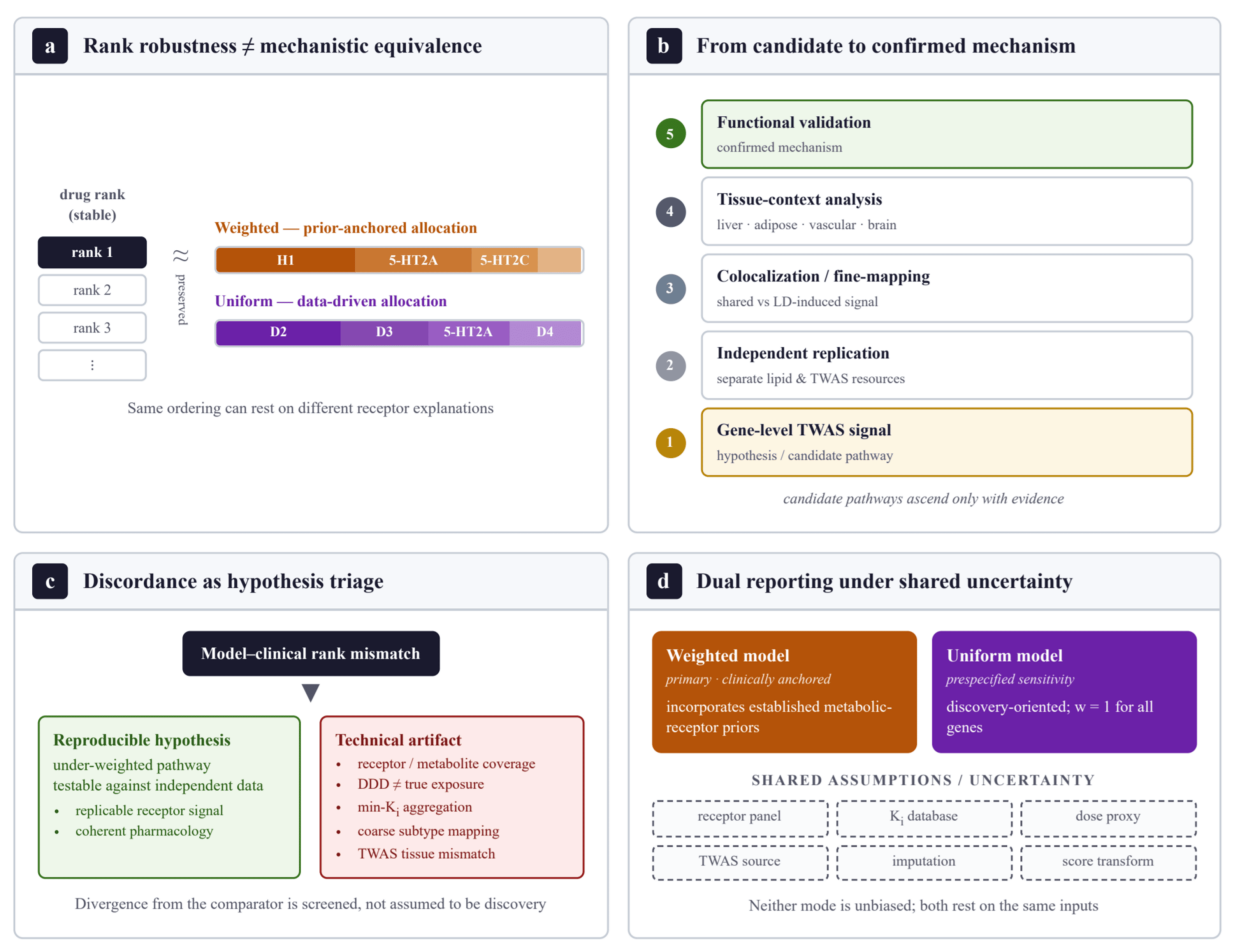
Conceptual structure of the discussion All panels are schematic illustrations of the interpretive framework. a) Rank robustness is not mechanistic equivalence. Removing literature-derived receptor weights largely preserves drug ordering (left), but reallocates the systems underlying each score: the weighted model concentrates signal in metabolic-risk receptors (H1, 5-HT2A, 5-HT2C), whereas the uniform model shifts it toward dopaminergic systems (D2, D3, D4). Thus, prioritization can be robust while biological interpretation remains sensitive. b) Evidence ladder from candidate to mechanism. A gene-level transcriptome-wide association indicates a candidate pathway, not a confirmed mechanism. Promotion requires replication, colocalization or fine-mapping to distinguish shared from linkage-disequilibrium signals, tissue-context resolution, and functional validation. Adrenergic and other low-prior candidates occupy the lowest rung. c) Discordance as hypothesis triage. Mismatches between model-derived and comparator clinical ranks are screening devices, not tests of clinical truth. Each is partitioned into biological hypotheses (an under-weighted but testable pathway) or technical artifacts from receptor/metabolite coverage, the defined-daily-dose proxy, minimum-Ki aggregation, coarse subtype mapping, or transcriptomic tissue mismatch. d) Dual reporting under shared uncertainty. The weighted model is primary; the uniform model is a prespecified sensitivity analysis. Neither is inherently unbiased: uniform weighting imposes equal contribution, and both depend on shared choices - receptor panel, Ki database, dose proxy, TWAS source, imputation, and score transformation - shown as the shared uncertainty layer. Bar segments, ladder rungs and node sizes are illustrative, not measured quantities. Credits: Ngo Cheung TWAS, transcriptome-wide association study; Ki, inhibition constant; DDD, defined daily dose; w, receptor weight; LD, linkage disequilibrium; H1, histamine-1; 5-HT, serotonin; D2/D3/D4, dopamine subtypes

The weighted analysis is therefore more clinically anchored, but its receptor interpretation is partly informed by prior assumptions. The uniform analysis is less dependent on those assumptions, but it is not inherently unbiased. Uniform weighting imposes its own assumption: that all recognized receptor genes should initially contribute equally. The two modes should thus be treated as complementary analyses rather than as a biased model and an unbiased model (Figure 2). The dual-reporting approach is proposed as a transparent internal sensitivity strategy; it has not been externally validated and should not be presented as an established clinical reporting standard.

ADRB1 as a high-priority computational candidate

ADRB1 was the most striking low-weight candidate. Its HDL TWAS z-score of 13.53 was substantially larger than the absolute z-scores of the other candidate genes, while its literature weight was only 0.08. The result is biologically plausible at a broad pathway level because adrenergic signaling and lipid regulation have been linked in prior physiological and genetic studies, but this plausibility does not establish an antipsychotic-specific mechanism.

Recent clinical-genetic studies of psychotropic-associated lipid change and antipsychotic-induced metabolic phenotypes also demonstrate that lipid-related genetic signals require replication, outcome-specific analysis, and independent cohorts before they can be used for clinical inference [5,24,25].

The present result should nevertheless be described as a candidate pathway rather than a confirmed mechanism. The ADRB1 signal is a gene-level TWAS association, not a direct experiment showing that antipsychotic binding to beta1-adrenergic receptors changes HDL. The direction of the HDL association is unknown in this analysis; the signal could correspond to higher or lower genetically predicted expression associated with higher or lower HDL. In addition, the same ADRB1 association was applied across multiple drugs, meaning that repeated drug-level hits do not constitute independent replication.

Several analyses would be needed before ADRB1 could be promoted from hypothesis to mechanism. The association should be replicated in independent lipid datasets and ideally in independent TWAS resources. Colocalization or probabilistic fine-mapping should assess whether the HDL association and the ADRB1 expression signal are driven by the same variant or a shared regional signal. Tissue-specific analysis should determine whether the relevant regulatory context is liver, adipose tissue, vascular tissue, brain, or another tissue. Finally, functional studies should evaluate whether changing ADRB1 expression or signaling alters lipid-related phenotypes under conditions relevant to antipsychotic exposure.

Clinical discordance as hypothesis generation

The discordance analysis should be viewed as a method for identifying mismatches, not as a direct test of clinical truth. Paliperidone was the largest mismatch, with model ranks of 35 under weighting and 41 under uniform weighting compared with a comparator rank of 4. Quetiapine, lurasidone, amisulpride, and cariprazine were also generally assigned numerically lower positions by the model than by the comparator.

Several explanations are possible. The receptor database may not represent all relevant targets or metabolites. Defined daily dose may not adequately represent clinical exposure. Selecting the minimum Ki may overstate some interactions and understate others. Receptor subtype mapping may be too coarse. The lipid TWAS inputs may not capture the relevant tissues or regulatory mechanisms. Some apparent clinical effects may also be mediated by pathways that are not represented in the current receptor-gene dictionary.

Ziprasidone illustrates the opposite pattern. It rose to an especially prominent position under uniform weighting, reaching a mean rank of 2.80 across traits in the V4 uniform model. This could reflect strong dopamine-related or broader receptor engagement that was down-weighted in the literature model, but the result does not show that ziprasidone has unrecognized clinical metabolic toxicity. It identifies a computational configuration that merits follow-up, particularly if it can be tested against independent receptor, lipid, or clinical data.

The fact that the model did not simply reproduce the supplied comparator is potentially useful for method evaluation. However, divergence can arise for both scientifically interesting and technically undesirable reasons. Because the comparator and model contain different drug sets and measure different constructs, the present discordance values should be treated strictly as hypothesis-triage devices rather than evidence of unrecognized clinical risk.

Value of dual reporting

Reporting both weighted and uniform models provides a transparent compromise between prior knowledge and discovery. The weighted model incorporates established pharmacological evidence and is easier to interpret in relation to known antipsychotic metabolic mechanisms. Its emphasis on HRH1 and HTR2C is consistent with receptor-based literature and previous analyses of weight gain and metabolic effects [6,10,8].

The uniform model provides a sensitivity analysis that removes differential receptor weighting. It reveals whether receptor systems with lower prior weights, including dopamine and adrenergic genes, could become important when judged by the observed affinity and TWAS data alone. This mode is best described as discovery-oriented rather than clinically neutral because it still depends on the selected receptor panel, Ki database, dose proxy, TWAS source, missing-data procedures, and score transformations.

For reporting within this computational study, the weighted V4 model can be presented as the primary clinically anchored sensitivity mode, while the V4 uniform model can be presented as a discovery-oriented comparator. This is a proposed internal reporting framework, not an externally validated clinical method.

The pooled receptor contribution figure should show both modes side by side. The ADRB1 candidate analysis and leave-one-gene-out results should be presented as hypothesis-generating computational analyses rather than as evidence of confirmed causal pathways.

Limitations

The first limitation is the absence of external clinical calibration. The model was not tested against longitudinal weight gain, lipid change, incident diabetes, metabolic syndrome, hospitalization, or cardiovascular outcomes. The clinical discordance analysis used a comparator ordering encoded in the supplied analysis, but it did not estimate predictive accuracy. The resulting score should therefore be described as a relative computational prioritization index rather than as a clinical risk estimate.

Second, defined daily dose is a harmonization tool, not a direct measure of patient exposure. It does not capture prescribed dose, treatment duration, adherence, pharmacokinetics, plasma concentrations, receptor occupancy, or active metabolites. The same defined daily dose may correspond to different biological exposures across drugs and patients.

Third, the minimum-Ki aggregation method selects the strongest observed measurement for each drug-receptor pair. This may be useful for identifying possible high-affinity interactions, but it can overemphasize an isolated experiment or a measurement obtained under conditions that differ from other assays. Ki values can also vary across experimental platforms, assay systems, receptor constructs, species, laboratories, and definitions of binding affinity. Affinity should not be treated as a direct substitute for receptor occupancy or tissue-specific pharmacodynamic effect. Median, mean, assay-stratified, or hierarchical aggregation should be evaluated in future sensitivity analyses.

Fourth, receptor mapping is simplified. Generic labels such as alpha-1, alpha-2, or muscarinic were assigned to representative subtypes. The mapping of 5-HT3 to HTR3A and generic muscarinic labels to CHRM1 may not fully represent the underlying pharmacology. These receptor-to-gene assignments were investigator-defined for the supplied pipeline and were not independently adjudicated. Receptor mapping uncertainty should be retained explicitly rather than hidden within a single deterministic dictionary.

Fifth, missing-value imputation may alter the score. Mean Ki imputation creates estimated affinities for receptor rows without direct measurements. TWAS imputation and default handling can provide comparability across drugs, but they may also reduce genuine heterogeneity. The supplied downstream outputs do not provide exact counts of observed, imputed, or default values by drug, receptor, and trait. This incomplete missingness quantification is a central reproducibility limitation rather than a minor reporting detail.

Sixth, nominal TWAS significance was defined as a p-value less than 0.05. This threshold was used for the drug-level significance bonus and was not adjusted for the number of genes, receptors, traits, drugs, or methods. It should therefore be regarded as exploratory. A confirmatory implementation should use false-discovery-rate or familywise-error control and should distinguish observed from imputed TWAS values.

Seventh, the V4 transformation uses negative log10 p-values and ignores association direction. Absolute z-score approaches also ignore direction by design. These transformations therefore assume that positive and negative gene-trait associations contribute similarly to the model score. That assumption may be inappropriate when higher and lower expression have opposite biological consequences. This directionless TWAS treatment is a central methodological limitation, particularly for HDL, for which the sign of the association is essential to interpretation. No direction-aware sensitivity analysis was performed.

Eighth, TWAS associations can be influenced by linkage disequilibrium and correlated expression-prediction models. Fine-mapping work demonstrated that LD can induce associations at noncausal genes, while formal colocalization is needed to distinguish shared from distinct signals in the same region [18,19]. The ADRB1 result should therefore be followed by colocalization, fine-mapping, replication, and functional testing.

Ninth, the scoring coefficients were heuristic. The logarithmic transformation, exponential p-value scaling, cap at 25, maximum-z multiplier, and nominal-significance multiplier were not optimized against clinical outcomes. The perfect reconstruction correlation confirms that the formula was implemented consistently; it does not validate the parameter choices. A systematic parameter-range analysis remains necessary.

Finally, the supplied code is a notebook export rather than a packaged workflow. It includes repeated function definitions, hard-coded paths, shell commands, and several versions of the scoring function. Although the Stage 2 reconstruction was faithful to the saved rankings, clean execution from a fresh environment remains important. Reproducibility should be established with a versioned repository, locked dependencies, unit tests, configuration files, input checksums, and independent reruns.

Intended use

The intended use of the framework is limited to relative computational prioritization, model-sensitivity assessment, and early pharmacological hypothesis generation. It may help identify drugs or receptor-gene features for replication, pharmacological follow-up, or assay design. It should not be used to estimate an individual patient’s metabolic risk, select an antipsychotic, guide monitoring frequency, or infer the direction of a lipid outcome. Any clinical use would require independent outcome validation, calibration, discrimination assessment, prospective evaluation, and assessment of generalizability across populations and treatment settings.

Translational potential

The framework may be useful for prioritizing drugs and receptor pathways for further pharmacological study. A weighted analysis can identify whether a drug’s score is dominated by established metabolic-liability receptors, while a uniform analysis can reveal receptor systems that might otherwise be suppressed by prior weighting. The comparison is particularly useful during early drug development, where both known liabilities and under-recognized pathways are relevant.

The candidate list could also guide targeted follow-up. ADRB1 is the most immediate candidate because of its unusually strong HDL-associated TWAS signal. CHRM4, DRD4, ADRB2, SLC6A4, and DRD2 provide additional targets for replication. Follow-up studies should evaluate receptor expression, tissue context, ligand pharmacology, direction of effect, and clinical lipid outcomes rather than relying on the computational score alone.

A longer-term application could involve patient-specific or drug-specific metabolic risk modeling. Such models would require clinical outcomes, treatment duration, dose, co-medication, demographic factors, baseline metabolic status, and genetic information. The present framework could contribute receptor and pathway features to such a model, but it cannot replace outcome-based validation.

Relationship to previous work

The weighted contribution pattern is consistent with earlier receptor-based work implicating H1, 5-HT2C, alpha-adrenergic, muscarinic, and related systems in antipsychotic metabolic effects [6,8,10]. The uniform model extends the interpretation by showing that dopamine and other broad receptor systems become more prominent when prior metabolic weights are removed.

The TWAS component follows the logic of PrediXcan, summary-based TWAS, and MetaXcan-style integration, but the present study adds a pharmacological layer that is not present in standard gene-trait association analyses [13-17]. The Stage 2 analysis therefore occupies an intermediate position between receptor pharmacology and genetically informed gene prioritization.

The work also extends the earlier method-comparison analysis of the integrated Ki-DDD-TWAS pipeline. That analysis found that alternative TWAS scaling methods produced highly concordant drug rankings, while the present analysis shows that removing receptor priors changes the model-attributed receptor composition more substantially than changing the scaling function alone [20]. This distinction should be emphasized: TWAS transformation sensitivity and prior-weight sensitivity are related but separate sources of model uncertainty.

Recent pharmacogenomic studies of antipsychotic-associated lipid and metabolic changes further support the need for independent replication and outcome-based validation before computational candidates are translated into clinical claims [5,24,25].

## Conclusions

This secondary computational sensitivity analysis demonstrates that the V4 Ki-DDD-TWAS framework is relatively robust at the level of broad drug ranking but sensitive at the level of model-attributed receptor contribution. Reconstructing the saved ranks produced a Spearman correlation of 1.000 across all five traits and both weighting modes, confirming implementation-level fidelity of the downstream calculation. The literature-weighted model concentrated pooled contribution in HRH1, HTR2A, and HTR2C, whereas the uniform model shifted emphasis toward DRD2, DRD3, DRD4, and other broadly engaged or low-prior receptor genes. This reallocation occurred despite a mean V4 rank correlation of 0.913 between modes. Clozapine remained the highest-ranked drug across all traits and modes, while several drugs, particularly ziprasidone, haloperidol, amisulpride, olanzapine, quetiapine, and paliperidone, showed meaningful differences in computational position. These positions are relative prioritization indices based on saved computational outputs and should not be interpreted as patient-level metabolic outcomes or estimates of clinical metabolic risk. ADRB1 was the strongest low-weight TWAS candidate, with an absolute HDL z-score of 13.53 and a predefined literature weight of 0.08. The result is biologically plausible at the level of broad adrenergic and lipid-regulatory pathways but remains hypothesis-generating. It requires independent replication, direction-aware analysis, colocalization or fine-mapping, tissue-specific assessment, and functional validation.

The framework is therefore a computational prioritization and sensitivity-analysis framework that has not been externally validated. It does not predict patient-level metabolic outcomes, does not estimate clinical metabolic risk, and cannot establish receptor-level causality. Receptor attribution remains hypothesis-generating, and prospective biological and clinical validation is required before any translational or clinical interpretation.

## Appendices

Appendix 1: Literature basis for the receptor-weighting scheme

The proposed receptor weights are best understood as literature-informed heuristic values, not experimentally measured effect sizes. The available studies support the general ranking of several receptors, but they do not demonstrate that HRH1 = 1.00, HTR2C = 0.92, or CHRM3 = 0.85 are quantitatively exact. In the current version of the pipeline, these weights are also inactive because USE_METABOLIC_WEIGHTS = False; all recognized receptor genes are assigned a uniform weight of 1.0.

The strongest support is for H1/HRH1, 5-HT2C/HTR2C, and M3/CHRM3. H1 affinity showed the clearest association with short-term antipsychotic-related weight gain, and H1 receptor occupancy was also strongly correlated with both weight gain and diabetes risk [6,10]. 5-HT2C antagonism is linked to impaired satiety signaling, increased appetite, and weight gain, making the high HTR2C value reasonable as a relative ranking [26,27]. M3 is especially relevant to insulin secretion and diabetes: olanzapine and clozapine inhibited cholinergic-stimulated insulin release in pancreatic islets through muscarinic M3 antagonism [27,28]. However, the exact ordering among these three receptors remains uncertain because H1 and muscarinic receptor occupancy are highly correlated across antipsychotic drugs [10].

The intermediate weights for HTR2A, ADRA1A/ADRA1B, and HTR6 are also defensible, although the evidence is less consistent. 5-HT2A antagonism has been associated with weight gain and impaired insulin responses, while α1A affinity was significantly related to short-term antipsychotic-associated weight gain [6,26]. Evidence for HTR6 comes from both receptor-binding analyses and more recent animal work showing that selective 5-HT6 ligands can modify antipsychotic-related changes in weight, glucose, and lipid measures [26,29]. The shared weight for ADRA1A and ADRA1B should nevertheless be described as an α1-family approximation, since most of the direct evidence concerns α1A or the receptor family rather than ADRA1B specifically.

The remaining weights should be treated more cautiously. D2 signaling is clearly involved in appetite, reward, prolactin, glucose regulation, and pancreatic function, but D2 antagonism alone does not explain why antipsychotic drugs differ so substantially in metabolic risk [26,27]. The α2 subtypes may have mixed or even protective effects, so assigning them uniformly positive weights is provisional. Similarly, the evidence for M1, HTR1A, HTR7, D3, D4, M4, M5, β-adrenergic receptors, SIGMAR1, and the monoamine transporters is not strong enough to justify precise independent coefficients [26,30,31]. Overall, the dictionary is most defensible as a transparent prior emphasizing H1, 5-HT2C, and M3, with the other receptor values regarded as exploratory rather than validated clinical risk estimates.
